# Regenerative Medicine: Advanced Therapy for Muscle Tissue Restoration

**DOI:** 10.3390/ijms27114762

**Published:** 2026-05-25

**Authors:** Roman Deev, Evgeniy Kopylov, Iurii Slepov, Nikita Gladyshev, Igor Limaev, Irina Sorochanu

**Affiliations:** A.P. Avtsyn Research Institute of Human Morphology State Scientific Center of the Russian Federation B.V. Petrovsky Russian National Research Center of Surgery, Moscow 117418, Russia; romdey@gmail.com (R.D.);

**Keywords:** sarcopenia, muscle trauma, gene therapy, cell therapy, orphan disease, inflammation, regeneration, tissue engineering, myogenesis

## Abstract

Skeletal muscle loss resulting from traumatic injury, sarcopenia, and myopathies remains a major clinical challenge due to the limited regenerative capacity of adult muscle tissue. This review systematically examines advanced biomedical therapeutic approaches to restoring muscle mass and function, including gene therapy, microRNA, cell-based strategies, and tissue engineering. Key mechanisms of muscle histogenesis and regeneration are discussed, with emphasis on the roles of satellite cells, growth factors (IGF-1, VEGF), and transcriptional regulators. Preclinical studies demonstrate that viral and non-viral delivery of myogenic factors can enhance muscle repair, reduce fibrosis, and improve functional outcomes. However, translation to clinical practice is hindered by challenges such as immune responses, inadequate reinnervation, and the complexity of replicating native tissue architecture. Emerging strategies combining gene delivery with rehabilitation, immunomodulation, or exosome therapy show synergistic effects. Although clinical trials targeting sarcopenia and muscle defects using anti-myostatin antibodies, stem cell-derived products, and acellular scaffolds have reported modest gains in strength and lean mass, no definitive regenerative therapy has been approved. While significant progress has been made, achieving full structural and functional muscle regeneration will require combinatorial approaches that address vascularization, innervation, and the inflammatory microenvironment.

## 1. Introduction

Over the last decade, the loss of muscle tissue has become a global challenge [1,2,3]. This may be due to the aging global population, with an increasing incidence of sarcopenia; the increasing number of combatants and injuries caused by high-energy weapons as a consequence of war and terrorist attacks; and the increased detection of genetically determined muscle deficiencies. Despite some advancements in the study of muscle histophysiology and the management of its individual elements [4], the information accumulated to date requires critical analysis.

Data in the literature indicate a high failure rate of medical interventions for musculoskeletal pathologies, including infections, loss of function, and the need for repeat amputations [5]. Following gunshot and mine-blast injuries, muscle tissue often suffers significant volume loss, necrosis (primary defect), and atrophy, leading to incomplete functional recovery even after reconstruction. Muscle loss is exacerbated by secondary injury and atrophy due to ischemia, fibrosis, and microcirculatory impairment, leading to disability in 40–60% of cases [6]. Reconstructive surgery primarily restores the functionality of injured limbs. After gunshot wounds, the risk of long-term disability is twice as high as with accidental injuries [7].

Age-related sarcopenia develops gradually and progressively. It is a syndrome characterized by loss of muscle mass, leading to impaired function as a result of chronic low-intensity inflammation, mitochondrial dysfunction, altered hormone secretion, and other factors [8,9,10]. Patients with sarcopenia are more likely to fall and suffer fractures, and the condition itself is a factor in adverse outcomes in malignant disease and chronic kidney disease [11,12]. The prevalence of sarcopenia varies considerably, affecting 5–13% of people aged 60–70 years, and 11–50% in people over 80 years. Rates are higher in certain settings; for example, up to 60% of patients in rehabilitation units and institutionalized elders suffer from sarcopenia [13,14].

A separate group of primary and secondary myopathies causes the loss of skeletal muscle. In the pathogenesis of Duchenne muscular dystrophy, rapid muscle loss leads to death due to respiratory failure and infectious complications. A number of other diseases with a longer course also result in muscle loss; the most significant of these conditions are limb-girdle muscular dystrophies, facioscapulohumeral myopathies, and other myopathies [15].

Tissue engineering may offer a means of restoring lost muscle volume [16]. However, despite the wide variety of methodological approaches to selecting scaffolds and populating them with muscle cells, an effective approach that ensures the implantation of a tissue-engineered construct into the recipient’s body and its subsequent proper functioning is still lacking, although experiments have demonstrated activity-dependent innervation of the transplanted construct [17,18,19]. In this regard, reconstructive and plastic surgery methods based on the non-free movement of tissue flaps, and even allogeneic muscle transplantation, remain relevant in medical practice [20]. These methods are characterized by a wide range of organizational (the search for donor material; ethical issues) and medical (e.g., immune rejection reactions, infectious inflammation) problems [21]. Despite the obvious need to develop advanced biotechnological methods to compensate for muscle tissue deficiency, these technologies remain in their infancy. This is partly due to the complexity of myohistogenesis, the natural physiological and reparative regeneration of striated skeletal muscle tissue, which is a multiphase process regulated by genetic and epigenetic factors.

The objective of this review is to summarize and present data on the initial results of experimental developments aimed at muscle recovery.

## 2. Regenerative Medicine and Muscle Tissue

### 2.1. Basic Concepts of Histogenesis and Regeneration of Muscle Tissue

Skeletal muscles are formed through the sequential connection and differentiation of the cellular embryonic myotome, which is a derivative of the mesoderm [22]. Subsequent cellular events leading to muscle formation include several key stages: the fusion of myoblasts with the launch of myotubes (activation of the genes *ITGB1*, *ITGA4*, *CAV3*, *FOXO1*, *NFATC2*), the synthesis of proteins of the contractile apparatus—sarcomere proteins—and their accumulation with the formation of large myosymplasts (Table 1) [23]. These integrate, forming a group of definitive muscle fibers which are subsequently innervated, leading to the formation of motor units. Importantly, some myoblasts are not included in the differentiation process and are preserved, especially in the parafibrillary niche between the basal membrane of the muscle fiber and the sarcolemma. These cells are called ‘satellite cells’, and at least some of them can be considered stem cells of muscle tissue. The presence of these cells allows skeletal muscle tissue to be classified as regenerative, meaning that in adults, there are cells that retain the potential for cellular reparative regeneration [24]. In the event of damage, these cells can enter the mitotic cycle and differentiate into myotubes, thus allowing the formation of new muscle fibers. Furthermore, it has been proven that they can fuse with a nearby muscle fiber, allowing it to share their nuclei [25].

**Table 1:** Key stages of myohistogenesis.

Myogenesis Stage	Gene	Protein	Functional Meaning	Reference
Migration of myoblasts from the myotome	*CXCR4*	Chemokine receptor CXCR4	Directs myoblast migration through chemotaxis	[26]
	*ITGB1, ITGA4*	Integrins (β1, α4)	Mediate adhesion to the extracellular matrix; necessary for directed migration	[27]
Myoblast fusion	*MYMK*	Myomaker (transmembrane protein)	The transmembrane protein required for the initiation of myoblast fusion	[28]
	*DOCK1*	Dedicator of cytokinesis 1	Activates Rac1 to remodel actin during cell fusion	[29]
Formation of muscle tubes	*MyoD*	Myogenic differentiation 1	Determines cell fate in the myogenic direction; activates genes encoding contractile proteins	[30]
	*MYOG*	Myogenin	Regulates cell differentiation at later stages than MyoD; activates contractile protein genes and completes differentiation	[31]
Synthesis of contractile proteins	*MEF2*	Myocyte enhancer factor 2	Transcription factor; interacts with MRFs to activate genes encoding contractile proteins (myosin, actin)	[32]
Innervation of muscle fibers	*AGRN*	Agrin	An extracellular matrix protein that induces neuromuscular synapse formation.	[33]
	*NRG1*	Neuregulin 1	A signaling protein; essential for the formation and function of the NMJ	[34]
Prevention of apoptosis of myoblasts and satellite cells	*BCL2*	B-cell lymphoma 2 (anti-apoptotic)	Inhibits apoptosis; localized on the mitochondrial membrane.	[35]

There are a few obstacles to the formation of full-fledged muscle tissue from this cellular progenitor cell reserve in an adult organism. Firstly, it has been established that the number of these cells decreases with age in several animals and humans [36,37,38]. They are likely expended over the organism’s lifetime to ensure physiological regeneration, and in the case of inflammatory or genetic diseases, to ensure repair. In addition, cellular aging and apoptosis reduce the resources of muscle progenitor cells, both under normal conditions and during the development of various diseases. For example, the expression of the *DLL1* gene, which maintains satellite cell populations and preserves their ability to proliferate, decreases [39]. This may occur due to chronic inflammation, decreased sensitivity to auto- and paracrine signals, cellular aging, and other causes, which contribute to a progressive decrease in the effectiveness of regeneration and the loss of muscle mass in sarcopenia [40,41]. Secondly, in cases of extensive or long-term muscle damage, likely due to a pro-inflammatory microenvironment, connective tissue cells that actively produce collagen matrix gain a particular morphogenetic advantage [42]. Their activity leads to fibrosis of the damaged area. Histologically, this is accompanied by atrophy and death of regenerating muscle fibers during muscle repair.

Thirdly, muscle tissue and its fibers are mechano-dependent structures that lack automaticity [43]. Their functional activity requires somatic innervation, which ensures the formation of neuromuscular synapses and the inclusion of formed fibers into motor units. The absence of innervation or aberrant interaction between cells leads to apoptosis or other types of cell death in muscle and prevents the formation of a “spatial regeneration template” [44,45,46].

### 2.2. Extensive Trauma

Traumatic injuries to skeletal muscles result from direct impact. Direct injuries occur under the influence of external mechanical forces causing contusions and/or ruptures of muscle tissue, accompanied by variable levels of necrosis. High-energy trauma, such as that resulting from car accidents, modern gunshot and/or mine-blast wounds, and crush syndrome, can lead to particularly extensive muscle defects. Significant muscle loss can result from surgical procedures. Indirect trauma occurs without direct external impact and is associated with functional or structural disturbances in muscle tissue, leading, for example, to excessive eccentric muscle contractions during intense physical activity [46]. In this case, the endogenous regenerative potential is insufficient to restore the structural integrity and functional activity of the skeletal muscle. This leads to the formation of coarse fibrous scars and permanent functional deficits, necessitating the search for exogenous methods to induce reparative myoregeneration [47].

**Figure 1:**
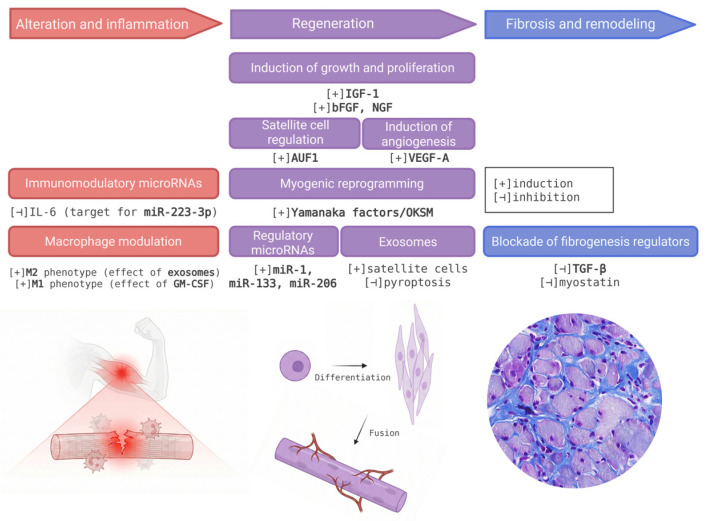
Directions for gene therapy research in traumatic muscle injury at different stages of healing.

Healing of damaged skeletal muscle is a complex biological process that occurs in several sequential and partially overlapping phases: alteration and degeneration, inflammation, regeneration, and fibrosis [48]. Each of these stages is a potential target for therapeutic development, but in the context of gene therapy and other Advanced Biomedical Therapy (ABMT) approaches, the regeneration phase and subsequent fibrosis are of greatest interest (Figure 1).

In traumatic injury, muscle tissue regeneration is ensured by satellite cells located between the basement membrane and the cell membrane of the fibers, the proliferation and differentiation of which ensures the formation of new muscle tissue and the restoration of damage [49,50,51]. This process is regulated by numerous mechanisms, including transcription factors (MyoD, Myf5, Mrf4, Mef2s), growth factors (FGFs, NGF, IGF), and microRNA (miRNAs), which coordinate the sequence of events necessary for the effective recovery of muscle tissue [23]. However, the basic unit of normal regeneration is not an individual myocyte but a niche comprising: a mature muscle fiber, its basal lamina, satellite cells, interstitial stromal cells, blood vessels, macrophages, and the neuromuscular junction [52].

Following the identification of the key growth factors involved in muscle tissue regeneration—basic fibroblast growth factor (bFGF), insulin-like growth factor 1 (IGF-1), and nerve growth factor (NGF)—accumulating evidence has continued to refine our understanding of their individual contributions to the regenerative process [37].

#### 2.2.1. Extensive Trauma and Gene Therapy

Weintraub et al. (1987) demonstrated the fundamental possibility of reprogramming fibroblasts in the myogenic direction (in vitro) without the fusion effect, which opened the era of developing methods for the gene induction of rhabdomyogenesis gene cell therapy [53]. Studies have long demonstrated the possibility of gene delivery in vivo, by direct administration of adenoviral vectors, and ex vivo, by transplantation of transduced myoblasts isolated from the hindlimb muscles of H-2Kb tsA58 transgenic mice [37,52]. Later, similar results were obtained in other preclinical studies using adeno-associated viruses (AAV) as a delivery system. The constructs increased muscle mass, tetanic force, and the number of Pax7-positive fibers in C57BL6 mice in a model of skeletal muscle atrophy following long-term immobilization of healthy hind limbs using plaster casts [54,55].

A combination approach—exercise and gene therapy using IGF-1 and recombinant AAV as a delivery system—also demonstrated a significant synergistic effect, doubling the gain in hindlimb muscle mass in Sprague Dawley rats compared to a no-exercise group. This strategy also slowed down muscle atrophy after exercise cessation [56]. This makes the combination of gene therapy and rehabilitation promising not only for accelerating muscle tissue regeneration but also for preventing secondary complications such as muscle loss due to reduced functional activity [57,58].

Given the effects of IGF-1, such as activation of proliferation and suppression of apoptosis in cells of various tissues, the search for alternative methods of targeted delivery of this gene is an important task. One of the proposed methods is non-viral delivery of plasmid DNA encoding the *IGF-1* gene using electroporation, which ensures local production of the therapeutic protein directly in the damaged muscle (injury–repair model BALB/c and C57BL/10 mice) [59,60]. Studies in 2006 showed that this method is superior to systemic administration of IGF-1 due to the more efficient transfection and increased levels of growth factor in the target tissue in mouse model of tibialis anterior muscles myotoxic injury [61]. IGF-1 is a potent stimulator of muscle growth and inhibitor of atrophy. Its AAV-mediated expression is able to counteract the atrophic processes induced by denervation by activating the Akt/mTOR signaling pathway and suppressing the expression of key regulators of atrophy, such as Atrogin-1 and MuRF1 [62].

Gene therapy using the vascular endothelial growth factor A (VEGF-A) gene delivered by AAV holds significant potential for the treatment of skeletal muscle injury, particularly in ischemic conditions where improved perfusion is critical for regeneration. In a rabbit study, AAV-VEGFA165 provided a sustained increase in muscle perfusion up to 7-fold compared to control (AAV-LacZ) in ischemic limbs, with an effect lasting up to 12 months [63]. However, unregulated long-term VEGF-A expression resulted in adverse effects, including aberrant vascular structures within muscle fibers (positive for CD31), increased extracellular matrix accumulation with macrophage infiltration, and fibrosis, which may impair muscle morphology and functional activity. In addition to growth factor studies, recent work focuses on regulators of muscle regeneration that are more fundamental (Table 2).

**Table 2:** Approaches of ABMT to induce muscle tissue regeneration.

№	Factor	Experimental Model	Result	Reference
Myospecific protein genes				
1.	Follistatin (*FS344*)	Healthy animals (NHP)	Increased quadriceps girth, increased fiber diameter, improved strength	[64]
Growth factors				
2.	IGF-1	Atrophy by immobilization (C57BL/6 mice)	Increased muscle mass, increased strength	[54]
3.	IGF-1, NGF, bFGF	Contusion of the gastrocnemius muscle (mouse)	Increase in the number and diameter of regenerating myofibrils	[53]
4.	VEGF-A	Limb ischemia (rabbits)	7-fold increase in perfusion	[63]
miRNAs				
5.	miR-1, miR-133, miR-206	Muscle injury (rats, Sprague-Dawley)	Activation of Pax7, MyoD1, and myogenin; inhibition of myostatin. Increased myofibril diameter, increased muscle strength.	[65]
6.	miR-223-3p	Muscle injury (miR-223-3p-deficient mice)	Uncontrolled inflammation, impaired regeneration and development of fibrosis	[66]
Other ABT approaches				
7.	Decorin (protein)	Rupture of the gastrocnemius muscle (C57BL/6 mice)	Increasing the proportion of regenerating muscle fibers and reducing fibrosis	[67]
8.	Exosomes (BMSC-Exos)	Contusion injury (mice)	Polarization of macrophages to the M2 phenotype, reduction of inflammation, improvement of regeneration	[68]
9.	*AUF1* (AAV8-*AUF1*)	Healthy animals (mice, C57BL6)	Satellite cell activation, reduction of atrophy markers, improvement of mitochondrial function, muscle growth	[69]
Inhibition of fibrosis				
10.	*GM-CSF*	Contusion injury (mice, C57/BL6)	Polarization of M1 macrophages, reduction of fibrosis, improvement of regeneration	[70]
Induction of innervation				
11.	HGF	Sciatic nerve injury, (mice, C57BL/6)	An increase in the thickness of the myelin layer, an increase in the diameter of axons, accelerates the transition of macrophages to the M2 phenotype	[71]
12.	GDNF		Increase in the thickness of the myelin layer	[72]
13.	*AGRN* (NT-1654)	SMAΔ7 mice	More mature neuromuscular junctions, reduction in muscle fiber thickness	[73]

Most of the key factors in satellite cell activation and maintenance are regulated by the AUF1 protein, which binds to untranslated regions of mRNA rich in AU repeats, causing their degradation or stabilization, thereby regulating gene expression [74]. Attempts have been made to increase AUF1 expression using gene therapy methods. The use of adeno-associated virus serotype 8 (AAV8-*AUF1*) for specific delivery of *AUF1* to the muscles of 12-month-old mice resulted in satellite cell activation, increased muscle mass, decreased atrophy markers, improved mitochondrial function, and improved oxidative capacity of muscle fibers in C57BL6 mice compared to the group injected with the virus containing the marker construct. It also increased the animals’ endurance during physical exercise. Additionally, knockout of the *AUF1* gene in 129/B6 mice resulted in hindlimb muscle atrophy compared to wild-type animals [69].

#### 2.2.2. miRNAs

Additional approaches to modulating muscle tissue regeneration include the use of microRNAs, particularly miR-1, miR-133, and miR-206, which regulate myoblast differentiation and proliferation [75]. In a model of surgically induced hindlimb injury in Sprague Dawley rats, muscle-specific microRNAs increased the expression of transcription factors involved in satellite cell proliferation and differentiation (Pax7, MyoD1, and myogenin), while inhibiting myostatin expression [65].

In addition to studying microRNAs that directly regulate myogenesis, some studies are aimed at investigating molecules that control inflammation [76]. An important component of complete reparative regeneration is the transition from the pro-inflammatory to the anti-inflammatory phase. As established by Cheng et al. (2020), miR-223-3p plays a key role in this process [66]. MicroRNA transported by immune cells to the site of injury acts as a negative regulator, suppressing the expression of the pro-inflammatory cytokine IL-6. Deficiency of miR-223-3p in mice resulted in uncontrolled inflammation, impaired regeneration, and the development of fibrosis. Therefore, local delivery of miR-223-3p or other immunomodulatory microRNAs may be an effective therapeutic strategy for optimizing the microenvironment and promoting muscle tissue recovery after injury.

#### 2.2.3. Antifibrotic Approaches

Inflammation, which occurs after both severe skeletal muscle injury and the systemic response of the organism, including sepsis, provokes a cascade of biochemical reactions leading to increased secretion of pro-inflammatory and profibrotic cytokines [77,78], among which proteins of the transforming growth factor beta (TGF-β) superfamily occupy the central place [79,80]. These molecules not only stimulate collagen synthesis by fibroblasts, forming scar tissue, but also directly suppress regenerative processes at the molecular level. Gardner et al. (2011) demonstrated that TGF-β inhibits autocrine production of insulin-like growth factor II (IGF-II) by myogenic precursor cells [81]. Thus, the use of TGF-β inhibitors aimed at stimulating endogenous IGF production or complex strategies combining IGF-1 delivery with TGF-β blockade may promote more effective healing and minimize fibrosis [82,83].

One of the representatives of the TGF-β superfamily is myostatin, a negative regulator of skeletal muscle growth which is actively involved in the development of fibrosis [84]. In in vitro experiments, myostatin stimulated fibroblast proliferation, promoted their transformation into myofibroblasts, and increased the production of TGF-β1 [85]. TGF-β1, in turn, induces myofibroblastic differentiation of myogenic cells in damaged tissues [86]. In addition to studies on the effects of known antifibrotic drugs such as interferon-gamma, relaxin, losartan, or monoclonal antibodies against TGF-β1, attempts have been made to study new molecules capable of suppressing TGF-β1 and myostatin [37,87]. One such molecule is the decorin protein, which neutralizes myostatin while stimulating follistatin production in fibroblast and myoblast cultures in vitro [85]. When the gastrocnemius muscle was ruptured in C57BL6J mice, the proportion of regenerating muscle fibers increased and the amount of fibrous tissue decreased with the administration of both decorin protein forms and with the use of AAV as a delivery system [67,88]. The use of the AAV1-*FS344* construct, encoding the natural myostatin inhibitor follistatin, contributed to an increase in the coverage of the quadriceps femoris muscle, the diameter of muscle fibers, and the strength of the limb into which the injection was administered, compared to the intact group in macaques [64].

Another mechanism for reducing fibrosis and enhancing myogenesis is the activation of M1 macrophages in the injury zone. The introduction of a plasmid vector transiently expressing granulocyte-macrophage colony-stimulating factor (*GM-CSF*) by electroporation shortly after contusion injury of the hindlimb muscles in C57/BL6 mice promoted the polarization of macrophages towards the M1 subpopulation, which reduced the severity of fibrosis and improved muscle fiber regeneration in the injury zone [70].

Macrophages are important regulators of muscle tissue healing through sequential polarization from the pro-inflammatory M1 phenotype to the anti-inflammatory M2 phenotype. In the early stages of injury (1–3 days), M1 macrophages initiate acute inflammation, promote the removal of necrotic debris, and stimulate the proliferation of myoblasts to a greater extent than fibroblasts. This creates optimal conditions for muscle tissue regeneration. M2 macrophages, which appear in the later stages of healing (4–7 days and beyond), facilitate the transition of the inflammatory process to the proliferative phase, suppress excessive inflammation, clear damaged tissue, and promote extracellular matrix remodeling [89].

#### 2.2.4. Transient Reprogramming In Situ

Methods for inducing pluripotency in previously differentiated cells for subsequent myogenic differentiation include the use of microRNA, Yamanaka factors (Oct4, Sox2, Klf4, and c-Myc-OKSM) [90], and alternative combinations of transcription factors [91,92,93]. However, long-term overexpression of transcription factors can lead to the uncontrolled proliferation of pluripotent cells, which is associated with the risk of developing tumors [94,95]. Transient and localized reprogramming of cells in situ by direct injection of plasmid DNA containing OKSM genes accelerates regeneration in a mouse model of surgical muscle injury [96]. A similar approach contributed to a decrease in fibrosis after injury in mice with the introduction of growth factors in situ, even with short-term expression of molecules, which was confirmed by histological examination [97].

#### 2.2.5. Exosomes

Another approach is the use of exosomes—extracellular vesicles that function as mediators of intercellular communication [98,99]. The therapeutic potential of exosomes in muscle tissue damage is due to their multifactorial action: they are necessary for the transport of proteins and regulatory RNAs, stimulation of satellite cell activation, modulation of the inflammatory response by polarizing macrophages to the M2 phenotype, and angiogenesis [100].

In 2021, Luo et al. (2021) [68] topically applied bone marrow stromal cell-derived exosomes (BMSC-Exos) to treat contusion injury in mice. Their therapeutic effect was found to be mediated by modulating the inflammatory response: exosomes promoted the transition of macrophages to an anti-inflammatory M2 phenotype, leading to reduced inflammation and improved regeneration. Subsequent work by the same group of scientists demonstrated that exosomes mediate intercellular communication between myoblasts and macrophages, regulating their interactions. Furthermore, current research is focusing on the creation of engineered exosomes selectively enriched for therapeutic microRNAs, which combines the advantages of both technologies [101].

Another area of exosome research is the treatment of ischemic muscle injury. Yan et al. (2020) [102] demonstrated that exosomes derived from umbilical cord mesenchymal stromal cells injected into damaged muscle effectively attenuate ischemic muscle injury caused by femoral artery ligation in C57BL/6 mice. The mechanism of this effect is based on the delivery of exosomal circular RNA (circHIPK3), which, upon penetrating target cells, inhibits miR-421, leading to increased FOXO3a expression. At the cellular level, it leads to the suppression of pyroptosis—a programmed inflammatory form of cell death—and a reduction in the release of pro-inflammatory cytokines IL-1β and IL-18.

Regardless of the specific mechanism—modulation of the immune response or direct prevention of cell death—the use of exosomes in injury models leads to the activation of a key regenerative program in muscle tissue. This is confirmed by the increased expression of the *MYOD*, *MYOG*, and *Pax7* genes, as well as improved muscle function in vivo [103,104,105].

### 2.3. Sarcopenia

As noted earlier, a decrease in the quantity and functional activity of the regenerative cell pool occurs because of the influence and interaction of many external and internal factors. These include a sedentary lifestyle, diet, imbalance in the secretion of pro- and anti-inflammatory cytokines, and anabolic hormones [106,107], which leads to a decrease in the regenerative potential of skeletal muscle tissue [107,108].

A change in the functional activity of the NMJ is one of the pathogenetic factors in the progression of sarcopenia [106]. A decrease in the efficiency of signal transmission through the synapse is due to a decrease in the number and activity of alpha motor neurons [109]. Denervation leads to fragmentation of the NMJ, which disrupts effective neurotransmission. Moreover, with age, the expression of nicotinic acetylcholine receptors on the postsynaptic membrane decreases, thereby contributing to the deterioration of signal transmission from the nerve fiber to the muscle [110]. There are currently no effective or specific methods for correcting this pathological condition, although a number of approaches are under investigation [111,112].

One therapeutic factor that promotes the restoration of quantitative and qualitative characteristics of muscle tissue is neurotrophin-3. It influences the mTOR signaling pathway, which plays a significant role in the process of protein synthesis in muscle tissue [113]. In addition to increasing the diameter of muscle fibers in the Charcot disease mouse model (B6.D2-Pmp22Tr-J/J), neurotrophin delivered locally as part of the rAAV1.NT-3 genetic construct at a dose of 1 × 10^11^ GC contributed to the improvement of signal transmission through the muscle synapse, prevented mitochondrial death, and enhanced their biogenesis [114,115]. Ozes et al. (2023) [108] evaluated the efficacy of NT-3 gene therapy in 18-month-old wild-type C57BL/6 mice, a model of natural aging and sarcopenia, by intramuscular injection of a viral vector (AAV1) with a muscle-specific promoter at a dose of 1 × 10^11^ GC. Gene therapy improved treadmill and rotarod performance, and contributed to the preservation of the myelin sheath thickness of nerve fibers and the number of neuromuscular synapses, according to histological and immunohistochemical studies. The authors also investigated the therapeutic potential of neurotrophin in a genetic model of accelerated aging SOD1^KO^ (superoxide dismutase deficiency). Mice were intramuscularly injected with the AAV1.tMCK.NT-3 vector at a dose of 1 × 10^11^ GC; 13 age-matched SOD1^KO^ mice without treatment served as a control. Therapy resulted in a significant improvement in physical activity: 3 and 6 months after gene therapy, an increase in the distance covered on a treadmill was observed, and the time spent on a rotarod increased by 30%. Treatment also contributed to an increase in the diameter of muscle fibers, especially fast-twitch ones [116].

Stem cell transplantation is studied as a potential treatment for sarcopenia, a condition characterized by a reduction in the number and functional activity of muscle stem cells. For example, transplantation of a pool of satellite cells expressing a unique combination of cell surface markers into the muscles of dystrophin-deficient mice (C57BL/10ScSn-Dmd^mdx^) promoted myofibril formation and restoration of dystrophin expression, significantly improving muscle contractile function [117]. Despite the potential of the muscle stem cell transplantation strategy to improve regeneration, their source is limited and in vitro cultivation is time-consuming and costly.

A possible method for obtaining myogenic cells is the stepwise induction of induced pluripotent stem cells (iPSCs) in vitro with regulatory molecules [118]. Methods for direct cell reprogramming (transdifferentiation) are under development, including melanocytes expressing Pax3 [119,120]. The possibility of using myogenic stem cells for skeletal muscle regeneration has been studied for a long time, but only limited successful results have been obtained [119,121]. Myogenic precursors obtained from induced iPSCs have a high proliferative potential and can be produced in significantly larger quantities compared to muscle stem cells. This allows for allogeneic transplantation, which in turn can contribute to more efficient cell self-renewal and muscle tissue regeneration in vivo. Various approaches to the generation of myogenic progenitor cells have been described in the literature, including gene overexpression methods and directed differentiation of iPSCs [122].

One method for correcting the state of reduced regenerative potential of muscle tissue is tissue engineering. This involves the use of various matrix materials—scaffolds in combination with muscle tissue stem cells and growth factors that stimulate myogenesis [123,124,125]. Tissue engineering of skeletal muscle aims to restore and/or regenerate muscle tissue using various cells, biomaterials, and bioactive molecules. Composite materials are being developed that incorporate natural and synthetic structures to achieve optimal physical, chemical, and biological properties and mimic the natural properties of muscle tissue. The main challenges of this approach currently stem from the presence of immune reactions, the need to ensure vascularization and innervation of the transplanted device, and the development of biomaterials capable of precisely regulating complex biological processes in regenerating muscle. All of this complicates the application of this technique in clinical practice [126].

### 2.4. Orphan Diseases and Chronic Inflammation

In the field of inherited muscle diseases such as muscular dystrophies, modern strategies, including gene therapy using AAV, have demonstrated significant progress [127,128]. However, their primary goal is to correct the primary genetic defect and, consequently, slow disease progression in relation to the remaining muscle fibers (MFs). These approaches are unable to replenish the already lost cell pool. The chronic cycle of degeneration and regeneration leads to the depletion of the cambial reserve of skeletal muscle tissue, represented by satellite cells, which ultimately limits the potential for both endogenous and therapeutically stimulated restoration [129]. This approach can only contribute to a limited histoprotective effect but does not restore already lost muscle volume.

In idiopathic inflammatory myopathies, the primary mechanism of damage is an autoimmune reaction directed against various components of the inflammatory myopathies. Regardless of the specific pathogenetic scenario—whether it be a humoral attack on the microvascular endothelium in dermatomyositis or a direct cytotoxic attack by T lymphocytes on the MF in poliomyositis—the result is chronic inflammation and persistent muscle fiber death. The death mechanisms here are more complex and include both necrosis caused by ischemia or the action of cytotoxic mediators and programmed cell death. Specifically, cytotoxic T lymphocytes can induce apoptosis in muscle fibers via the perforin–granzyme pathway. This also leads to the depletion of the regenerative pool and replacement of muscle tissue with fibrous tissue, making inflammatory myopathies another important target for regenerative approaches [130].

Muscle loss can also be secondary to damage to the innervating motor neuron or disruption of neuromuscular transmission. Denervation deprives the MFs of trophic support, triggering the intrinsic (mitochondrial) apoptotic cascade. Concurrently, protein synthesis sharply decreases due to suppression of the Akt/mTOR anabolic signaling pathway, and the cell’s primary catabolic systems, the ubiquitin–proteasome system and the autophagy–lysosomal system, are activated. This leads to a progressive decrease in MF diameter, which is morphologically manifested by the appearance of atrophic, angular variants [131]. If reinnervation does not occur within a certain timeframe, which in humans can range from several months to 1–2 years depending on the distance to the target muscle, atrophy becomes irreversible and culminates in MF death, followed by fibrosis. Thus, therapeutic strategies for neurogenic atrophy can be aimed at both restoring innervation and directly influencing muscle tissue to prevent its degradation [132].

### 2.5. Clinical Trials ABMT in Muscle Trauma and Sarcopenia

Recent clinical trials show promising advances in biomedical therapies for sarcopenia, focusing on muscle regeneration through pharmacological agents and multi-component interventions, though no drugs are yet approved (Table 3).

Several clinical trials in various stages are currently exploring interventions consistent with ABMT. NCT05211986 (IMM01-STEM), an open-label, dose-escalation phase I/II trial (recruiting), evaluates the safety and tolerability of IMM01-STEM (secretome from partially differentiated pluripotent stem cells) via twice-weekly intramuscular injections for 4 weeks in up to 18 patients with quadriceps atrophy due to moderate knee osteoarthritis (KL grade 2–3). Cohorts of test doses range from 225 μg to 2000 μg, monitoring dose-limiting toxicities, adverse events (CTCAE v5.0), knee strength (isometric torque), 6 min walk test, and WOMAC index up to 3 months post-treatment. Early data suggest good tolerability with no severe injection reactions; preliminary strength/function gains align with prior reports of 10–15% improvements [133]. Completed phase II trial assessed bimagrumab (anti-ActRIIB monoclonal antibody inhibiting myostatin/activin) in sarcopenia patients, showing significant lean body mass increases of 5–8% versus placebo at 16–24 weeks, with fat mass reductions. Secondary outcomes included improved 6 min walk distance and stair-climb power, though no FDA approval followed due to mixed functional endpoints. Safety profile was favorable, with mild GI events predominant [133].

MF-300 from Epirium Bio is an investigational oral inhibitor of 15-hydroxyprostaglandin dehydrogenase, designed to elevate PGE2 levels in aging muscle, enhancing muscle quality and strength independently of mass increases. MF-300 showed dose-dependent pharmacodynamic effects, including early and sustained reductions in urinary PGE2 metabolites (matching max muscle force gains in aged mice models) and increases in urinary PGE2 comparable to post-exercise human muscle levels, unlike placebo declines. Follow-on phase 1 data in older adults (January 2026) confirmed consistent metabolite reductions across participants. A positive FDA Type C end-of-phase 1 meeting (January 2026) aligned on phase 2b design for sarcopenia patients, including endpoints, dosing, and Fast Track potential.

In a 13-patient cohort study, implantation of acellular ECM bioscaffolds combined with early physical therapy for Volumetric Muscle Loss (VML) treatment led significant improvements in strength (37.3%) and range of motion (27.1%) at 6 months versus untreated controls, supporting ECM bioscaffolding as a viable regenerative approach for VML [134].

VA project I01RX00122204 (preclinical/early human) explores muscle stem cells, emphasizing bioengineered constructs’ need for exercise to optimize outcomes like force generation. There have been no large-scale recruiting phase II/III trials. Focus remains on safety and partial regeneration [135].

## 3. Future Directions

There are currently many advanced biomedical therapies, both experimental and clinical, targeting natural targets that can influence the sequential process of myohistogenesis to restore lost muscle tissue. Despite this relative diversity, the lack of convincing success in inducing histotypic muscle tissue regeneration motivates interest in broader generalizations based on the entire body of published data. Our AI query was directed to ChatGPT, model 5.2, extended thinking without memory. Important limitations were identified.

Prompt:

Imagine that you’ve just been activated. You have no memory of previous requests. Answer 3 questions about skeletal muscle gene therapy and sarcopenia.

Questions:

(1) Can gene-therapy interventions targeting muscle cells deliver full-fledged skeletal muscle regeneration?
(2) Which agents/targets/platforms in gene therapy are realistically needed for that (i.e., those that are likely to solve the problem, not “could in theory”)?
(3) Similar for sarcopenia: which gene-therapy approaches are genuinely capable of solving the problem? Mandatory requirements for the answer: Strictly critical stance: start with constraints and “why it may not work,” then “what still looks promising.”

Answer:

1. Full skeletal muscle regeneration (fascicle architecture, reinnervation, vascularization, long-term force) is poorly reducible to deliver a gene into a myofiber. In an acute, large-volume defect, the matrix and cellular sources are missing; even perfect growth-factor expression does not recreate the spatiotemporal assembly of tissue and the NMJ, and the risk of fibrosis/contractures remains. In chronic dystrophies, tissue is often partially preserved and the task is closer to “fix a protein/pathway,” so transferring solutions from acute trauma is limited. In sarcopenia, the problem is systemic (neuromuscular, inflammatory, metabolic), so “muscle-only” delivery rarely scales to functional restoration.

2. Realistic platforms: AAV (AAV9, AAVrh74, AAV1/6) for long-term expression, but constrained by immunity, unreliable re-dosing, cassette-size limits, incomplete tropism, and tight control of expression level. LNP/mRNA for short-lived pulses, but with poorer targeting to mature muscle and a need for repeat dosing; ex vivo (editing satellite cells/myoblasts followed by transplantation) is promising for local defects, but scaling and engraftment are weak. Targets: inhibiting catabolism (myostatin/activin–ActRIIB) more often yields hypertrophy without guaranteed strength and can worsen the tendon–muscle balance. Stimulating anabolism (IGF-1/PI3K–AKT) increases mass but carries risks of uncontrolled growth/metabolic effects. Regulating satellites/niche (PAX7/Notch/Wnt) runs into pool exhaustion and oncogenic risk under prolonged activation. Anti-fibrosis/inflammation (TGF-β/CTGF, macrophage switching) is highly timing-dependent and limited by systemic toxicity; NMJ (MuSK/agrin) requires coordination with motoneurons. Vascular component (VEGF/ANGPT) is limited by safety and vessel quality.

3. For sarcopenia, “curing” it with gene therapy is unrealistic: causes are multiple, and the effect must be titratable and reversible. The most plausible approaches are moderate, combined interventions: partial inhibition of the myostatin/activin axis (early clinical) to support strength/mass, but with uncertain long-term safety. Local, short-lived LNP/mRNA approaches to “prime” training responsiveness (data are scarce). Modulation of NMJ stability and oxidative phenotype (e.g., PGC-1α as a concept; predominantly preclinical) potentially closer to function, but without clinical proof.

Thus, the long-term forecast for the next 20–30 years suggests that the most likely scenario is improved clinical outcomes in certain forms of muscular dystrophy. This will be possible with gene replacement therapy technologies using AAV and genome editing methods, supplemented by adjuvant therapies aimed at slowing catabolic processes and suppressing fibrosis. In this context, defining the “therapeutic window”—the period during which etiotropic treatment should be initiated as early as possible, before irreversible muscle atrophy—is crucial. Regarding sarcopenia, the most realistic outcome appears to be partial correction of muscle weakness and increased exercise tolerance in certain sub-groups of patients, while achieving complete “reversible rejuvenation” is unlikely.

## 4. Conclusions

This review demonstrates that the restoration of lost skeletal muscle tissue remains an unresolved challenge across three distinct clinical contexts: VML following extensive trauma, age-related sarcopenia, and orphan myopathies. For each condition, a broad spectrum of ABMT approaches, including gene therapy, microRNAs, exosomes, anti-fibrotic agents, and transient reprogramming, has been explored. Nevertheless, no single modality has yet achieved complete histotypic regeneration. It is critical to recognize that full skeletal muscle regeneration entails not merely the recovery of tissue volume but the coordinated restoration of myofibers, the basal lamina and extracellular matrix, vascular network, neuromuscular junctions, and sustained force-generating capacity. In ordinary acute muscle injury, this goal is attainable to a substantially greater degree than in VML because the preserved basal lamina provides a spatial template that guides endogenous regeneration [136]. In VML, by contrast, the tissue scaffold and neurovascular integration are irrevocably disrupted. In chronic muscular dystrophies, the primary genetic or protein defect is compounded by secondary chronic inflammation, progressive fibrosis, and exhaustion or dysfunction of the satellite cell niche. In sarcopenia, the dominant pathophysiological drivers are age-related deterioration of the motor unit, fragmentation of the neuromuscular junction, and alterations in the metabolic and endocrine environment. Consequently, while gene therapy may serve as a central therapeutic module for selected monogenic myopathies, for VML and sarcopenia it will almost certainly remain only one component of combined protocols. More broadly, orphan myopathies illustrate that effective muscle repair depends not solely on myogenic cells but critically on the integrity of the extracellular scaffold and the three-dimensional tissue architecture, without which even normal myoblasts fail to restore muscle [137]. Thus, across all etiologies, the preservation or reconstruction of the spatial tissue template—along with immunomodulation, revascularization, and reinnervation—represents an essential, albeit not exclusive, prerequisite for advancing beyond partial repair toward functional muscle restoration.

## Figures and Tables

**Table 3:** Clinical trials of ABMT in sarcopenia and muscle trauma.

NCT/ID (Name)	Condition	Intervention	Phase/Status	Key Outcomes
NCT05211986 (IMM01-STEM)	Sarcopenia	Mesoangioblasts	I/II Recruiting	+10–15% strength
NCT01604408 (RESILIENT)	Sarcopenia	Bimagrumab (anti-myostatin)	II Completed	+5–8% lean mass
MF-300 (Epirium)	Sarcopenia	Novel therapy	I Completed 2025	Safety met, well-tolerated
Acellular ECM Scaffold	VML	Bioscaffold implant	Human cohort (13 patients)	+37% strength at 6 mo

## Data Availability

No new data were created or analyzed in this study. Data sharing is not applicable to this article.

## Abbreviations

iPSCs	Induced Pluripotent Stem Cells
MF	Muscle fibers
ABMT	Advanced Biomedical Therapy
VLM	Volumetric Muscle Loss
PGE2	Prostaglandin E2
ECM	Extracellular Matrix
AAV	Adeno-Associated Virus
GM-CSF	Granulocyte-macrophage Colony-Stimulating Factor
BMSC-Exos	Bone Marrow Stromal Cell-derived Exosomes
NMJ	Neuro-Muscular junction
